# False-Negative CSF Cryptococcal Antigen with *Cryptococcus gattii* Meningoencephalitis in Southeastern United States: A Case Report and Literature Review

**DOI:** 10.1155/2020/8232178

**Published:** 2020-07-13

**Authors:** Shawn Esperti, Austen Stoelting, Andrew Mangano, Dveet Patel, Jilian Sansbury, Robert Sherertz

**Affiliations:** Department of Internal Medicine, Grand Strand Health, Myrtle Beach, SC, USA

## Abstract

A 70-year-old immunocompetent male in South Carolina was admitted secondary to altered mental status and headache without focal neurological deficits. Head CT was negative. Lumbar puncture (LP) revealed normal glucose, elevated protein, and lymphocytosis. Opening pressure was 15 cm of H20. CSF lateral flow assay was negative for cryptococcal antigen; CSF cultures showed no growth. The patient rapidly improved on acyclovir and was diagnosed with presumed viral meningitis, as viral PCR and fungal culture were pending at time of discharge. The patient's condition quickly worsened and the patient returned one day later with right arm weakness and dysarthria. Brain MRI revealed T2/flair signal abnormalities in the left frontal lobe with associated parenchymal enhancement. Repeat LP revealed increasing white blood cell count with a worsening lymphocytosis and decreasing glucose, and opening pressure remained normal. CSF fungal culture from the first admission grew *Cryptococcus gattii*, and repeated CSF cryptococcal antigen and culture returned positive. The patient was started on IV steroids, induction Amphotericin and Fluconazole, followed by maintenance oral Fluconazole. The patient's clinical course was complicated by a brainstem lacunar infarction, which led to demise. We present this case of *Cryptococcus gattii* meningoencephalitis to highlight the risk factors, characteristics, and challenges in diagnosis and treatment of an emerging disease in the Southeastern United States.

## 1. Introduction

Cryptococcal infections have been well recognized as a serious infection of primarily immunocompromised patients worldwide with mortality rates up to 40% [1]. Cryptococcal organisms are often present in soil and infect the host through inhalation of spores with subsequent dissemination from the lungs to the brain [2]. Most cases of cryptococcal meningitis have been presumed *Cryptococcus neoformans,* as *Cryptococcus gattii* is rarely reported and historically only endemic to tropical regions [3]. Before 1999, clinical isolates of *C. gattii* in North America were almost nonexistent with a small number of cases reported in California and Hawaii [4]. Since 2004, multiple cases of human *C. gattii* infection have emerged in Oregon, associated with an outbreak on Vancouver Island and in mainland British Columbia, Canada [5]. Since this outbreak from 2005 to January 2013, 169 human cases of *C. gattii* infections were reported to the CDC with most confirmed cases in Oregon (88), Washington (31), California (28), Georgia (8), Florida (3), and one case each from Alabama, Colorado, Rhode Island, South Carolina, and Utah [6]. Despite the emerging number of cases, successful diagnosis and recognition remains a challenge. Historically, cryptococcal infections have been associated with HIV-infected patients, thus successful diagnosis has been especially challenging in immunocompetent patients. In addition, less is known about *C. gattii* infections compared to *C. neoformans*, and its incidence is likely underreported.

In suspected patients, the mainstay of diagnosis of *Cryptococcus* has become antigen immunoassays as they are highly sensitive, specific, and result quickly, while fungal cultures take days to weeks to grow [7]. The most widely used assays include the lateral flow assay, latex agglutination, and enzyme immunoassays. Lateral flow assays (LFA) are the most widely used due to objectivity, affordability, and turnover time [8]. A positive LFA is followed by the more labor intensive latex agglutination (LA) or enzyme immunoassay (EIA) to determine infection titers. Our lab used Immuno-Mycologics (IMMY) LFA, followed by Latex Agglutination System (CALAS; Meridian Biosciences). Despite such high sensitivity and specificity quoted of >99% in detecting CSF antigen for both the IMMY LFA and LA (CALAS; Meridian Biosciences), false-negative results have been reported [9–11]. We present this case of initial false-negative CSF cryptococcal antigen with *Cryptococcus gattii* meningoencephalitis to highlight the risk factors, characteristics, and challenges in diagnosis and treatment of an emerging disease in the Southeastern United States. We will also examine current antigen immunoassays used, pitfalls, and phenomena that can lead to rarely reported false-negative results with resultant delayed diagnosis and treatment.

## 2. Case

A 70-year-old male with a past medical history of low testosterone, hypertension, benign prostatic hyperplasia, and no known travel history presented with confusion and headache in South Carolina. The patient had been recently treated for community-acquired pneumonia and completed a 5-day course of amoxicillin/clavulanic acid as an outpatient. He presented 5 days later after developing a frontal headache and short-term memory deficits. Vital signs were significant for a fever of 100.4. Physical exam revealed lethargy without any focal neurological deficits. A CT scan of the head was normal. Lumbar puncture showed a cerebrospinal fluid (CSF) WBC of 103 K/mm^3^ with a differential of 55% lymphocytes, 7% neutrophils, 8% monocytes, glucose 56 mg/dL, and protein 180 mg/dL. Opening pressure was 15 cm H_2_O. The patient was started on vancomycin, ceftriaxone, ampicillin, and acyclovir for empiric treatment of meningitis and encephalitis. CSF studies were negative for cryptococcal antigen, Lyme IgM antibody, Toxoplasmosis IgG antibody, varicella, VDRL, and CMV. Viral HSV PCR was pending and bacterial gram stain, culture, and fungal culture revealed no growth on day 3 of hospitalization. The patient rapidly improved and was discharged with suspected viral meningitis on acyclovir.

**Figure 1:**
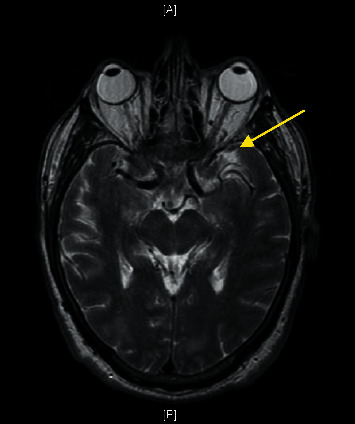
MRI brain with and without contrast showing diffuse leptomeningeal enhancement and several scattered foci of restricted diffusion, particularly in the left frontal and parietal lobes. There are some suggestions of T2/flair signal abnormality in the left frontal lobe with associated enhancement of the parenchyma, which could suggest early cerebritis (yellow arrow).

**Figure 2:**
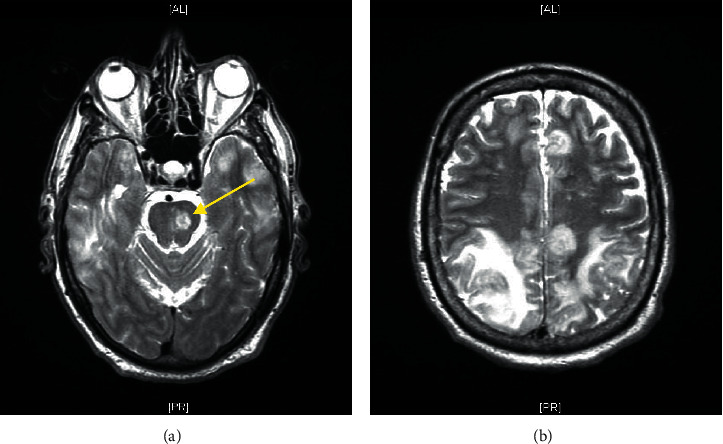
MRI brain with and without contrast showing (a) new left brain stem lacunar infarct (yellow arrow) and (b) worsening sulcal and cisternal T2 hyperintensity and nodular pial enhancement suggesting progression of meningitis. New rather extensive symmetric vasogenic edema due to progressive cerebritis.

The patient returned to the hospital one day after discharge with new onset right-sided weakness and dysarthria. MRI of the brain revealed T2/flair signal abnormalities in the left frontal lobe with associated parenchymal enhancement (refer to Figure 1). Repeat LP was performed and CSF showed a WBC of 621 mg/dL with a differential of 85% lymphocyte, 29% PMNs, 16% monocytes, CSF glucose 21 mg/dL, and CSF protein 127 mg/dL. Opening pressure was 19 cm H_2_O. CSF fungal cultures from the previous admission grew *Cryptococcus gattii* after 5 days. HIV and hepatitis B and C were negative; serum IgA, IgM, and IgG levels and ANA were normal. Repeat CSF antigen was obtained but not resulted, and gram stain and bacterial cultures showed no growth. Fungal cultures grew *C. gattii* in 3 days. The patient was started on induction therapy of IV liposomal Amphotericin and Fluconazole for four weeks. A third LP was performed after two weeks of induction therapy with LFA-confirmed positive cryptococcal antigen and a titer of 1 : 2560 on LA. There was no growth on fungal culture. Repeat weekly LPs showed decreasing CSF antigen to 1 : 80 titers and continued sterile fungal cultures. The patient was transitioned and discharged on oral Fluconazole for maintenance therapy. Ten days later, the patient returned to the hospital after suffering a large brainstem lacunar stroke (refer to Figure 2). He was transitioned to inpatient hospice and later expired.

## 3. Discussion

Since the British Columbia outbreak in 1999, the *C. gattii* has become more prevalent in the United States with the CDC reporting cases in Oregon, Washington, California, Georgia, Florida, Alabama, Colorado, Rhode Island, South Carolina, and Utah [6]. Despite the recent emergence of *C. gattii*, infections from this species are still likely underreported compared to *C. neoformans*. While *Cryptococcus* more often infects immunocompromised patients, *C. gattii* has shown to have a much stronger affinity to affect immunocompetent patients [3]. Many centers have identified an increasing number (up to 20%) of cases of *Cryptococcus* in immunocompetent patients [12]. Clinicians should be suspicious of patients presenting with signs of meningitis who are from potentially endemic regions such as the Southeastern United States or those who have traveled to these areas.

Excluding HIV, other comorbid conditions have been associated with increased risk of *C. gattii* infections. Immunocompromised hosts include those with solid organ transplants, hematologic malignancies, other malignancies, rheumatic diseases, and those receiving immunosuppressive therapies including corticosteroids and TNF-alpha inhibitors [13]. Immunocompetent hosts at the highest risk are patients with chronic diseases such as cirrhosis, chronic lung disease, chronic renal insufficiency, sarcoidosis, and diabetes mellitus. Once *C. gattii* infection has been established in an HIV-negative patient without the aforementioned risk factors, less common forms of immunosuppression should be considered and evaluated if feasible. *C. gattii* infection has been associated with patients with autoantibodies to GM-CSF, hyper IgM/IgE disorders, lymphoproliferative disorders, CD4+ T-cell lymphocytopenia, and usage of monoclonal antibodies, specifically infliximab, etanercept, adalimumab, and alemtuzumab [14].

Prompt recognition and treatment of central nervous system infections related to C. *gattii* is crucial, as there are several potentially devastating neurological complications. The most common is elevated intracranial pressure and hydrocephalus [15]. Opening pressure should be obtained at initial diagnostic LP and at two weeks when repeat fungal cultures are obtained [16]. If the patient develops signs and symptoms of elevated intracranial pressure, serial LPs and ventriculoperitoneal shunt may be indicated [17, 18]. A complication of chronic cerebral meningitis is lacunar cerebral infarctions. Infection can lead to vasculitis, inflammation-induced vasospasms, and thrombosis presenting as lacunar infarctions often in the thalamus, internal capsule, basal ganglia, and rarely the brainstem, as occurred in this patient [19].

The LFA, LA, and EIA have been shown to have sensitivities and specificities as high as 100% in detecting cryptococcal antigen in the CSF [20]. A recent study using 51 archived CSF samples compared the performance detection of 4 immunoassays: Meridian EIA, LA test, IMMY EIA, and IMMY LFA. Among the 51 CSF samples, 18 were positive and 33 were negative and the results showed 100% agreement among positive (18/18) and negative (33/33) samples for each assay [20]. Despite the success of immunoassays have had in detecting *Cryptococcus* CSF antigen, data on *C. gattii* immunoassays are minimal and sample size is small. False-negative testing with the IMMY LFA assay from the prozone effect has been documented [20, 21]. The prozone effect is often observed in the presence of a high antigen titer as in this patient who had titers of 1 : 2560 after two weeks of treatment [22]. When a large antibody response is mounted, antibodies bind to the cryptococcal antigen, leaving no antigen for the IA to detect, resulting in a false-negative test [22]. In addition, low fungal burden in localized diseases such as isolated cryptococcoma and acapsular cryptococcal variants can lead to false-negative antigen tests, thus delaying diagnosis [23]. In this case, delayed diagnosis may have been avoided if the LFA or LA were repeated rather than anchoring on viral meningitis. Due to pitfalls and potential false-negative results of immunoassays, clinicians should consider repeat cryptococcal antigen testing in both immunocompromised and immunocompetent individuals in endemic areas.

## 4. Conclusion

*Cryptococcus gattii* infection should be highly considered in patients presenting with meningitis who reside in or have traveled in geographic areas with known cases, even in the absence of underlying immunosuppression. Despite the success immunoassays have had in detecting Cryptococcus CSF antigen, data on *C. gattii* immunoassays are minimal. False negative detections, as in our patient's case, can lead to delayed diagnosis and poor outcomes. This case highlights the risk factors, characteristics, and challenges in diagnosis and treatment of *Cryptococcus gattii* meningoencephalitis in the Southeastern United States.

## Data Availability

No data were used to support this study.
